# Requirement Analysis of Different Variants of a Measurement and Training Station for Older Adults at Risk of Malnutrition and Reduced Mobility: Focus Group Study

**DOI:** 10.2196/58714

**Published:** 2024-09-17

**Authors:** Lisa Happe, Marie Sgraja, Vincent Quinten, Mareike Förster, Rebecca Diekmann

**Affiliations:** 1 Junior Research Group “Nutrition and Physical Function in Older Adults” Department of Health Services Research Carl von Ossietzky Universität Oldenburg Oldenburg Germany

**Keywords:** gerontechnology, physical activity, diet, technical assistance system, health data, qualitative research

## Abstract

**Background:**

Demographic change is leading to an increasing proportion of older people in the German population and requires new approaches for prevention and rehabilitation to promote the independence and health of older people. Technical assistance systems can offer a promising solution for the early detection of nutritional and physical deficits and the initiation of appropriate interventions. Such a system should combine different components, such as devices for assessing physical and nutritional status, educational elements on these topics, and training and feedback options. The concept is that the whole system can be used independently by older adults (aged ≥70 years) for monitoring and early detection of problems in nutrition or physical function, as well as providing opportunities for intervention.

**Objective:**

This study aims to develop technical and digital elements for a measurement and training station (MuTs) with an associated app. Through focus group discussions, target group requirements, barriers, and favorable components for such a system were identified.

**Methods:**

Older adults (aged ≥70 years) were recruited from a community-based setting as well as from a geriatric rehabilitation center. Focus group interviews were conducted between August and November 2022. Following a semistructured interview guideline, attitudes, requirements, preferences, and barriers for the MuTs were discussed. Discussions were stimulated by videos, demonstrations of measuring devices, and participants’ ratings of the content presented using rankings. After conducting 1 focus group in the rehabilitation center and 2 in the community, the interview guide was refined, making a more detailed discussion of identified elements and aspects possible. The interviews were recorded, transcribed verbatim, and analyzed using content analysis.

**Results:**

A total of 21 older adults (female participants: n=11, 52%; mean age 78.5, SD 4.6 years) participated in 5 focus group discussions. There was a strong interest in the independent measurement of health parameters, such as pulse and hand grip strength, especially among people with health problems who would welcome feedback on their health development. Participants emphasized the importance of personal guidance and interaction before using the device, as well as the need for feedback mechanisms and personalized training for everyday use. Balance and coordination were mentioned as preferred training areas in a MuTs. New training options that motivate and invite people to participate could increase willingness to use the MuTs.

**Conclusions:**

The target group is generally open and interested in tracking and optimizing diet and physical activity. A general willingness to use a MuTs independently was identified, as well as a compelling need for guidance and feedback on measurement and training to be part of the station.

## Introduction

### Background

As a result of demographic change, the proportion of older people (aged ≥67 years) in the total population in Germany is steadily increasing. It is predicted that, by 2040, a quarter of the total population will be older than 67 years. This demographic change is leading to a decline in the working population, including in the health and care sectors. In this context, new approaches to prevention and rehabilitation are needed to enable older people to live longer, more independent, and healthier lives [1,2]. This pursuit of longer independence and well-being is known as healthy aging and aims to maintain mental, physical, and social health for as long as possible. The 2 important elements of healthy aging are a balanced diet and regular physical activity. These 2 elements not only influence each other, but are also linked to other determinants, such as cognitive health [3]. Individuals aged >80 years, women, those with multiple comorbid conditions, and older adults living alone are at higher risk for malnutrition [4,5]. Among older people living in the community, the prevalence of being at risk of malnutrition, as measured by the Mini Nutritional Assessment or Mini Nutritional Assessment Short Form, is 19% and is significantly associated with reduced physical functioning, reduced independence in activities of daily living, and the presence of physical frailty and sarcopenia [6,7]. Low physical activity is generally associated with an increased risk of death; in particular, people who spend >10 hours a day sitting have a significantly increased risk of death [8]. For people with sarcopenia, that is a significant loss of muscle mass, function, or strength, or any combination, exercise training and nutritional interventions are among the most effective interventions to improve strength, walking speed, and balance [9]. In addition to structural and health system conditions, the health literacy of older people plays a crucial role in healthy aging. Although health literacy in Germany is somewhat higher than in other European countries, 54% of people aged >76 years have limited health literacy and may have limited knowledge about the importance of a balanced diet and sufficient physical activity in older age [10]. Older adults with low health literacy have been found to have lower levels of physical activity [11,12]. In the area of dietary adherence, current evidence from systematic reviews suggests that improving self-efficacy expectations is a key factor in the long-term implementation of healthy eating habits [12,13]. Accordingly, strategies such as psychological models should be used to increase participants’ self-efficacy expectations. To be able to respond to problems in these areas at an early stage, it would make sense to regularly check the nutritional and physical status of older people. In practice, however, such screening and advice are often not carried out due to a lack of resources and information about available interventions [14].

A technical assistance system could be an approach for the early detection of nutritional and physical activity deficits and the initiation of appropriate measures. Several approaches already exist to enable older adults to self-report nutrition and physical activity parameters. Parts of the geriatric assessment can already be automated [15-17]. In addition, there are programs that support older people to independently implement interventions to improve dietary and physical activity behavior [18-21]. However, most of these programs are interventions without automated assessment and are targeted at people with high functionality or specific medical conditions. A technical assistance system that can objectively measure the nutritional and physical activity status of community-dwelling older adults, as well as provide individualized feedback and training, could help to identify deficits at an early stage for secondary prevention, prevent negative health outcomes, and reduce the burden on the health care system.

However, before developing such a system, it is important to analyze in detail the general acceptance of older people for it, as well as their needs and their specific requirements for implementation and design.

### Aim

The aim of the study was to identify technical and digital elements that could be integrated into the technical assistance system to develop a system that is as effective and usable as possible. In addition, the target group’s requirements for the system and the general conditions for its use were determined.

## Methods

### Ethical Considerations

The study was approved by the ethical review board of the Carl von Ossietzky Universität Oldenburg (registration 2022-089). We conducted the study in accordance with the Declaration of Helsinki [22], as amended, and the underlying data protection regulation. Reporting followed the COREQ (Consolidated Criteria for Reporting Qualitative Research) checklist for interviews and focus groups [23]. All participants included in the study provided written informed consent.

### Participants

We recruited the participants from a geriatric rehabilitation center and in the community in the northwest of Germany.

Flyers were placed on the wards of the geriatric rehabilitation center and the electronic patient database was checked by a member of the study team for patients aged >70 years without known cognitive impairment. These patients were contacted and informed about the study.

Recruitment of the older adults in the community was done by placing flyers and organizing information sessions in various community-based organizations and settings, for example, sports groups for older adults, community or church-run public meeting places, and classes for older adults.

Interested people were approached in a face-to-face meeting to answer questions about the project and, if consent was given, to check the inclusion criteria. Inclusion criteria were (1) aged ≥70 years; (2) the presence of malnutrition according to the Mini Nutritional Assessment Short Form (0-7 points) or the presence of 1 or more risk factors for malnutrition: weight loss in the last 3 months, reduced food intake in the last 3 months, or a reduced BMI (<23 kg/m²) [24]; and (3) presence of 1 or more signs of reduced mobility (walking speed <0.8 m/s and Short Physical Performance Battery <8 points). Exclusion criteria were (1) lack of ability to give consent and (2) insufficient ability to understand the study content and procedure or the German language. Eligible persons were interviewed for sociodemographic data (sex and age), and technology commitment was assessed using a questionnaire by Neyer et al [25] with a 5-point Likert scale with 12 items about personal contact, interest, and general use of technology. A higher score indicates a higher technology commitment [25].

Once 6 suitable participants agreed and their eligibility was checked, a date for the focus group was arranged. All focus groups were held in person. To minimize the burden on the participants, the focus groups were held in the rehabilitation center, in the rooms of the respective sports club or senior citizens’ program, or the facilities of the study team, depending on the participants’ preference. There were no repeated discussions, and each participant was only allowed to take part in 1 focus group.

### Interview Guide

On the basis of the project objective, which was the development of a measurement and training station (MuTs) and corresponding app that could be used independently, key issues were identified for discussion in the focus groups. This was done through discussions within the project team involving people from different professions, such as nutritionists, physiotherapists, physicians, and computer scientists. For the selection of the health data and sensors to be presented, it was checked which possibilities for autonomous recording already exist and which parameters have already been identified in other work in other contexts as relevant for older adults, such as activity sensors, which can have a motivational effect to increase activity. In terms of training equipment, the aim was to represent different training areas and types of training. The areas chosen were endurance (bicycle ergometer), balance (oscillatory platform), coordination and cognition (exergaming system), strength training using body weight (3D depth image–based training correction), and everyday training (exercise stairs). The devices presented were also selected to ensure that independent training was possible. In the first phase of the focus groups (focus groups 1-3), these 5 training options were presented to the participants. In focus groups 4 and 5, only the 3 variants that had received the highest approval in the previous groups were shown. These were the oscillating platform, the exergaming system, and the 3D depth image–based training correction.

The guide was pretested with a pilot focus group of community-dwelling older adults and minor adjustments were made. The selection of devices was also discussed in the pilot focus group, and it was emphasized that the opportunity for participants to contribute their own experiences and wishes regarding measurement and training devices could and should be included in the discussions. The participants of the pilot focus group were not involved in the focus groups 1 to 5). The final interview guide covered 4 main topics in the first focus groups (focus groups 1-3) presented in Textbox 1.

Following the first 3 focus groups, the semistructured guide was adapted to obtain more detailed information on the previously identified relevant aspects. The revised interview guide used in focus groups 4 and 5 included 4 main themes presented in Textbox 2.

**Main topics covered in focus groups 1 to 3.**

**Relevant health data and measurement tools for independent use**

- Presentation of different health data and measurement devices
- Participants were asked to mark on paper with photos of the devices what they were interested in and what they could imagine using
- Open discussion on the participants’ choice

**Exercise or training options that are of interest for use in the measurement and training station (MuTs)**

- Presentation of different training devices through videos
- Each participant should enter numbers from 1 (most likely to be used in the MuTs) to 5 (least likely to be used) in an overview of training devices
- Open discussion about the participant’s choice

**Frequency and amount of time the participants were willing to spend in the MuTs**

- Brainstorming: Participants were asked to express their thoughts on the frequency of measurement and training, and statements were written on posters

**Using the MuTs and receiving instructions**

- Participants were asked to express their thoughts and needs, and statements were written on a large poster

**Main themes included in focus groups 4 and 5.**

**Usability of an electronic device for measuring handgrip strength (** **KForce Grip, Kinvent Biomecanique) and requirements for a corresponding evaluation screen**

- Demonstration of the handgrip strength measuring device and testing by participants
- Open discussion on thoughts and experiences during measurement
- Presentation of 3 different mock-ups of the grip strength feedback screen; each participant was given 3 printouts and asked to mark what they liked and disliked
- Open discussion on the comprehensibility of the mock-ups and ideas for improvement

**Abilities and requirements for self-administered questionnaires**

- Demonstration of how to complete the questionnaire on the tablet, with participants trying it out and watching a video of how to complete it on the screen

**Exercise or training options that are of interest for use in the measurement and training station**

- Presentation of different training devices through videos
- Brainstorming on the pros and cons of the training options

**Frequency and amount of time for additional training at home with a corresponding tablet**

- Demonstration of the exercise diary on the tablet for participants to try out
- Brainstorming: Participants were asked to express their thoughts on the frequency of measurement and training, and statements were written on posters

### Procedure

All focus groups were structured in a standardized way. First, the participants were welcomed and made comfortable with drinks and snacks. Then, a short input was given on the importance of a healthy diet and sufficient physical activity for older adults. The links between diet and physical activity were also discussed. This was followed by a presentation of the project idea of developing a MuTs, accessible to the public, where older people can independently measure their nutritional and physical activity status and have the opportunity to complete a training program and continue training at home using an app. A 3D model of a prototype of the MuTs was shown to give the first spatial impression of such a MuTs. Then, the interviewers introduced themselves and explained their role in the project. The participants were then asked to introduce themselves to the group.

The discussion started following the semistructured interview guide. For each topic, a short prompt was used, for example, showing videos of the use of the different training devices, and field notes were taken. To facilitate discussion, participants were also asked to mark their favorite aspects of health data, measurement devices, and training options with stickers or rank them in order of importance. After the semistructured guide was completed, participants were asked whether they wanted to add anything. Finally, the focus groups were closed by thanking the older adults for their thoughts and discussions. The transcripts of the focus groups were not returned to the participants for organizational reasons.

All interviews, except focus group 2, were conducted by the same 2 female interviewers together (LH and MS). The interviewers are a physiotherapist and a nutritionist working as scientific researchers in the field of technical assistance systems for nutrition and mobility in older adults. Focus group 2 was conducted by MS and VQ, who are a male physician and a computer scientist. At the beginning of each focus group, the aim of the project and the researchers involved with their expertise and credentials were introduced.

The focus group discussions were audio recorded. The length of the focus groups varied from an average of 76 (SD 6.2) minutes in the first 3 focus groups to an average of 96 (SD 11.5) minutes in focus groups 4 and 5.

### Analysis

The audio recordings were transcribed by 1 person using f4 software (f4, audiotranskription) in the form of an extended semantic transcription. When identifiable aspects were mentioned, this text was deleted and replaced by neutral words, for example, city. Pseudonyms were used to distinguish between the speakers. All transcripts were checked against the audio recordings by a study team member. A structured qualitative content analysis was conducted according to the concept of Kuckartz [26]. Computer-assisted coding was performed using MAXQDA 2022 software (MAXQDA Analytics Pro 2022; VERBI Software). Two members of the study team (LH and MS) derived main categories a priori from the interview guide. Subcategories were generated inductively during the coding process. LH and MS performed the coding process independently and then discussed the categories and coding critically until consensus was reached. Code saturation was checked by analyzing how many new categories could be found using additional focus groups [27]. The project’s participatory research council, consisting of older adults (aged ≥70 years), reviewed and critically discussed the material and categories for discrepancies in interpretation or understanding.

## Results

### Overview

A total of 21 older adults (female participants: n=11, 52%; mean age 78.5, SD 4.6 years) participated in the focus group discussions. In round 1, only 2 (33%) of the originally recruited 6 people participated in the first community-based focus group. It was, therefore, decided to conduct a second community-based focus group in this round with n=5 participants (42% of the total from the second round). Of the 9 older adults in the second round, 5 (55%) older adults participated in the community-based focus group and 4 (45%) in the rehabilitation focus group. The characteristics of the participants of the 5 focus groups are shown in Table 1.

The content analysis of the focus groups 1 to 3 revealed 6 main categories with 19 subcategories (Multimedia Appendix 1). After focus group 1, there were 17 (89%) subcategories out of 19 subcategories. The 2 new subcategories were both created after the analysis of focus group 2. Focus group 3 did not produce any new subcategories. In the focus groups with the revised interview guide, 5 main categories with 15 subcategories were found (Multimedia Appendix 2). In focus group 4, a total of 14 (93%) subcategories of the 15 subcategories were found in the focus group that took place in the rehabilitation center. Moreover, 1 additional subcategory was found in the community focus group (focus group 5).

**Table 1:** Overview of participants characteristics (N=21).

	Round 1			Round 2	
	Rehabilitation (n=5)	Community 1 (n=2)	Community 2 (n=5)	Rehabilitation (n=4)	Community (n=5)
Sex (female), n (%)	3 (60)	1 (50)	2 (40)	1 (25)	4 (80)
Age (y), mean (SD)	82.8 (3.1)	78.5 (0.5)	76.6 (3.6)	78.3 (4.0)	77.8 (5.1)
SPPB^a^ (score),mean (SD)	4.8 (1.3)	8.5 (0.5)	9.6 (1.7)	7.5 (1.8)	7.8 (2.2)
MNA-SF^b^ (score), mean (SD)	7.8 (2.8)	9.4 (1.9)	9.5 (0.5)	8.5 (3.5)	7.8 (1.0)
TC^c^ (score), mean (SD)	30.8 (8.8)	36.0 (1.0)	37.2 (5.1)	45.0 (6.0)	42.8 (8.6)

^a^SPPB: Short Physical Performance Battery.

^b^MNA-SF: Mini Nutritional Assessment Short Form.

^c^TC: technology commitment.

### Round 1

#### Health Data and Measurements

Participants expressed a general interest in different types of health data. Most of them were also very interested in self-measurement in the MuTs. Some health data were already regularly measured at home by older adults, such as blood pressure and the number of steps. They also mentioned that measuring health data is of special interest if they have a disease or health limitation related to these health data, especially in the discussion about cardiovascular diseases. The participants discussed that health data should be assessed every time they visit the MuTs, with some people also expressing concerns about measuring too much data because of the psychological stress that can be caused by a huge amount of different data and not listening to themselves and how they feel:

What’s the point of all these measurements and, um, these people wearing these watches and all that.... I want to live and not control myself all the time.

Female, 79 years

As different types of health data were shown and discussed, we found that handgrip strength measurement was largely unknown to our participants. After explanation, most participants expressed interest in this parameter. Different opinions were expressed regarding the assessment of oxygen saturation. Some participants were very interested and explained that this health data would be useful in combination with a fitness tracker to assess physical activity. However, some participants also had concerns about the interpretability of this data.

Measuring activity was relevant for almost all participants but more as an assessment in everyday life and not as part of every visit to the MuTs. Measuring activity during the visit to the MuTs was discussed, and it was expressed that it would be biased if activity was only measured during the visit. Some participants expressed that it should be combined with the collection of other health data, such as pulse or oxygen saturation.

The evaluation of cardiological health data in the MuTs was discussed intensively. Participants were especially interested in pulse and blood pressure data and mentioned that these are the data that most of them measure at home. In particular, people with cardiovascular disease expressed a general interest in this type of health data. Other cardiological data, such as electrocardiography (ECG) and heart sounds, were described as data that should be assessed and evaluated by physicians and not as part of MuTs:

Um, and ECG less, because that’s a story that I think is a bit more difficult in terms of effort and too many mistakes can be made. Um, I think it’s very difficult when there are methods that are easy, ok, but yes.

Male, 77 years

#### Using Measuring Devices

The handling of the different devices (blood pressure monitor [wrist and upper arm], fitness tracker [wrist and clip], and hand dynamometer) was described as relatively easy by all participants, with the exception of the upper arm blood pressure monitor, where participants reported difficulties with the readability of the display. Some devices were already used regularly by participants, most commonly blood pressure monitors (wrist) and fitness trackers. People who already used devices also reported varying degrees of difficulty or compliance with regularly monitoring their blood pressure, and some mentioned that they did not rely on the results. The use of a home emergency call system was raised as an issue by the participants, and including some kind of emergency call system in the MuTs was seen as highly relevant. In general, participants expressed a preference for devices that can measure multiple health outcomes and include some form of direct data transfer to the MuTs:

But if I could go to the station and wear this watch and it would immediately measure my steps, pulse and oxygen. That would be very good. So, for me, the difference is that if I go there specifically, I can use more complex things, they give me more.

Female, 78 years

#### Physical Training Variants

To ensure a good training environment, several aspects should be met. These include the relevance of the exercises to everyday life (eg, steps should have real heights, and the training should aim to make everyday life easier and should be individually tailored to the problems of the participants). Another important aspect that was frequently mentioned was safety during the training. For example, the risk of falling should be reduced using handrails.

Although familiar devices would be easier to use, they would be less attractive if they were already available at home. Sports clubs are visited by some participants; further training should only take place if training is not available at the sports club. Instructions from the physician about which sports to avoid are taken very seriously. An advantage of the MuTs over training at home is that there is space for all the training equipment and the availability of new, additional training equipment that participants may not have at home.

Training should challenge different areas and be adapted to the ability of the user. In general, training balance to prevent falls, training reaction time, cognition, and muscle strength for improve everyday activities were described as important. Training should also be fun, enjoyable, and motivating. An insight into one’s own past results would encourage ambition; comparison with the training results of others was rejected. An overly technical look of a training device could be a deterrent.

An overview of the participants’ comments on the different training devices is given in Multimedia Appendix 3. Overall, devices for training coordination and balance were rated higher than those for training endurance. In addition, devices offering training options that are less available in everyday life were preferred.

#### Range of Use

Almost all participants emphasized that the use of the MuTs should not restrict other areas of life. In many cases, other activities would already compete with the use of the MuTs and take up more time in the lives of older people. In addition, the participants emphasized that self-motivation, the distance to the MuTs, and the travel possibilities had a great influence on the extent to which the station would be used:

And I would, um... once a week, because I also want to do other things than for my body, I also want to do something for my mind and for my social life. And I also need time for that. And of course, I need more time for everything than I used to. You have to go slower to get there...yes, everything takes more time.

Female, 78 years

One participant expressed that the MuTs should not replace medical examinations but only support or relieve the physician at times when there are no serious health problems.

Many participants stated that they would be guided by official recommendations for use, such as information on the frequency at which training begins to have a positive effect, or how often to measure which health parameter. It was generally stated that the MuTs would not be used spontaneously but would have to be planned in advance by the participants. Participants indicated that they would like to stay between 15 and 90 minutes. Participants most often indicated that 30 to 45 minutes would be appropriate, that a shorter duration would not be worth the journey, and that a longer duration would reduce strength and concentration.

#### Expectations and Requirements for Using the MuTs

All participants considered human involvement to be an important prerequisite for the use of the MuTs. The initial introduction should always be given by a person, and there should also be the possibility to contact this person in the course of the use of the station if there are any uncertainties or problems. Aspects mentioned regarding the use of the MuTs instructions for measurement or training were asking questions, repeating instructions, or skipping instructions if they were already known. Disadvantages mentioned in the course of use due to the lack of a real trainer were that the station does not provide social interaction to alleviate loneliness and that interaction with a real trainer would lead to more fun, better results, and higher motivation:

I have problems with voices talking to me and telling me what to do. That it’s not a person at all. So, for example, with this instruction of what to do, um, it wasn’t clear to me when I was watching, when she said it, should I do it right away or should I listen to it all together and then do it?... And that’s when a certain aggression starts to develop against this machine voice.

Female, 78 years

If the health parameters measured in the MuTs deteriorated significantly, a physician should definitely be involved, or the person should be advised to have the change checked out by a physician. Particularly in the case of cardiological measurements, such as an ECG, there was little confidence in a measurement taken on the station, and the importance of involving a physician was highlighted.

The general usability of a touch system to control the MuTs is considered to be good and is already known from other situations (supermarket checkout). Short and simple sentences would be important for the usability of the MuTs, as well as sufficient volume for the audio output to enable people with hearing impairments to use them. For structuring purposes, information should be broken down into small action steps, as it is feared that comprehension may be impaired in the MuTs due to anxiety, excitement, fatigue, or pain.

### Round 2

#### Measuring Handgrip Strength via an Electronic Measurement Device

Although there was no prior knowledge or expectation of handgrip strength measurement, the participants showed great interest and understanding of the relationship between overall muscle status and the measured value. After an explanation of the handgrip strength parameter and its significance, many participants emphasized that they would find regular monitoring useful. The fact that the measurement required little equipment and was quick and not very strenuous was rated positively. Several participants described the process of measuring handgrip strength as logical or not difficult. Opinions differed as to whether instructions should be given once before the first measurement or before each measurement. Options for this were written or audio instructions:

And I think it’s really good when you’re guided through it. So, I start and then it says “next” and then the next picture comes up and then there’s the hint again. Something like I have to press again or I have to press now. So, I think that explains it quite well at the beginning because you can see it.

Female, 74 years

Many participants emphasized that it should be easy to log in for measurement. A familiar system such as ID cards should be used for registration, or access data should be entered manually.

#### Presenting and Interpreting Results of Handgrip Strength Measurement

The focus groups generally showed a good understanding of the various examples of graphs showing the results of the grip strength measurements used to stimulate discussion. In general, the diagrams should be short and clear, have strong contrasts, and not be overloaded with too much information (especially text). All participants were in favor of including a cut-off value in the figures so that they could interpret whether their own handgrip strength corresponded to the corresponding standard values and, if necessary, estimate the amount of deviation. An overview of previous measurements would be important to be able to assess one’s own development over time.

A graph showing a comparison with other study participants was discussed intensively. Most participants were against a comparison. The comparison was not relevant to them, not interesting, and not very meaningful:

That’s the thing, I have too much information, I don’t need it. I want to know my results.

Male, 73 years

#### Answering Questionnaires on a Touch Screen

To stimulate discussion, participants were shown how to complete a questionnaire on a tablet in the session and a video of the questionnaire being completed on a 139.7 cm touch screen. A general comment was that it would depend on the prior technical knowledge of the participants as to whether problems would arise in completing digital questionnaires. If there was little prior knowledge, an introduction to the technology would be helpful. Further support should be provided depending on the technical skills of the participants:

I would have to learn how to deal with it. It’s a foreign object to me.... But I would learn, probably.... I would want to learn it, yes.

Female, 74 years

The font size of the questions on the tablet was found to be easy to read. However, the legibility on the screen at the station was even better. The advantages of completing the questionnaire at the station were the better clarity and larger font size. Filling in the questionnaire was said to be quicker because everything was immediately visible and there was no need to scroll. Other participants suggested that it was quicker to complete the questionnaire on the tablet. It was also noted that the time window for completing the questionnaire on the tablet was flexible.

Contextual factors were particularly criticized when the questionnaire was completed at the station. The main concern was that the time spent at the station to complete the questionnaire would be significantly longer and that the user would be under time pressure from waiting participants. The perceived time pressure would lead to a lack of concentration and influence response behavior. Another concern expressed by 1 participant was that the data entered could be seen by other participants and that privacy could no longer be guaranteed. It was also criticized that completing the questionnaire in the MuTs was less convenient than at home and that it did not make sense to go to the MuTs just to complete the questionnaire there:

You can sit down, you can sit down quietly and concentrate.

Male, 73 years

#### Physical Training Variants

In general, all 3 training options (oscillatory platform, exergaming system, and 3D depth image–based training correction) were well received, as shown in Multimedia Appendix 3. The exergaming system received the most positive feedback and generated the most interest. The various advantages and disadvantages of the 3 training options presented and discussed are summarized in Multimedia Appendix 3. In this focus group phase, the participants also discussed which form of presentation of the 3D depth images they would prefer when using the camera system. The color image was the preferred form of presentation, as the sequence of steps in the exercise is easier to recognize and the exercise is shown more clearly due to the higher contrast.

In general, the adaptability of the training to different performance levels would be important. Regardless of the type of training, many participants considered balance training to be very important and some also considered coordination training to be very important. Participants described how older people were more likely to use familiar equipment. New equipment that requires extensive instruction was more likely to be rejected. Complicated equipment could also be overwhelming for older people. Equipment with multiple components, a lot of technology, or a high susceptibility to faults was rated as complicated. Experiences were reported that frustration quickly sets in, and successes become less visible:

Especially the potential for failure. The more you build in, isn’t it, the more you run the risk of failing again in the whole story. The simpler it is, the less you can have a mistake somewhere in there.... That also frustrates you. You also want to see some success, or it’s not like that.

Male, 83 years

#### Continuing Exercises at Home

As part of this discussion, a digital training plan for the autonomous performance of simple physical exercises at home was shown and found useful by the participants. The training plan itself and the symbols used (exercise duration and number of repetitions) were understood by all participants. The font size and readability were also rated positively. One person with little experience of using tablets was initially very reluctant to use the device and expressed concern about breaking something.

The desired frequency of use per week varied considerably. Most participants favored a training frequency of 2 to 3 times per week, assuming a training duration of 20 to 30 minutes per session.

One of the most frequently mentioned factors influencing the implementation of home exercise was how it could be integrated into the participants’ daily lives and how flexible it would be. Both the physical condition of the participants and their own sporting activities or social life were seen as competing with a home exercise program. Adequate space or exercises that do not take up much space would also be a prerequisite for exercising at home. Motivation to exercise also depends on whether success is visible, whether the exercises are variable, and whether exercising has already become an integral part of the participant’s everyday life:

You might have to move a carpet or two to avoid falling. But that’s, that’s quite feasible. So, if you...seriously want to do it, you’ll find a way.

Male, 79 years

## Discussion

### Principal Findings

The aim of the study was to identify technical and digital elements that could be integrated into the technical assistance system to develop a system that is as effective and usable as possible. In addition, the target group’s requirements for the system and the general conditions for its use were determined.

We were able to conduct 3 focus group discussions with older people living in the community and 2 focus group discussions in a geriatric rehabilitation center. Our focus group discussions revealed general interest and openness among the target group to track and optimize nutrition and mobility independently.

When discussing relevant health data and the competence of older people to record it independently in the MuTs, we observed that the willingness and interest to perform measurements seemed to be greater, especially if there was already a health limitation. Similar observations were made in the study by Bian et al [28], which investigated the use of a home sensor system for frailty diagnosis. People who described themselves as healthy and physically active saw less sense in using certain sensors. In the study by Seinsche et al [29], willingness to use an exergame-based telerehabilitation device was evaluated by focus groups. They found that the willingness to use technical devices with a meaningful purpose was even more pronounced than the general willingness to use technology. This demand for a perceived benefit from the use of an assistive technology system had already been observed in an earlier focus group study with older people [30].

Participants in our focus groups were mostly positive about the use of technical measurement devices to collect health data. They were confident enough to use almost all the devices presented independently after receiving instruction. The fact that it often seems to be a prejudice that older people tend to have problems with technology has also been shown in other qualitative studies conducted with older people on the development of MuTs [28,29,31]. The studies by Bian et al [28] and Seinsche et al [29] describe that older people have few problems using technology and are interested in modern technologies. They note that it is particularly important to build confidence and self-efficacy in using technology and that general knowledge of how to use it is usually less of a problem.

Possibly, another factor contributing to the acceptance of automated health records is the age of the study participants. The average age of the participants in our study was 78.5 years. This makes them slightly older than participants in other publications (average age 71.3-76.6 years) on older people’s willingness to use and perception of autonomous technical assistance systems in the area of nutrition and activity [28,29,32]. Our study and the other aforementioned studies showed a general willingness to use such systems. On the other hand, a study of people aged >85 years in a nursing home showed that these people were less interested in monitoring their health status [33].

People using the MuTs should also be able to get direct feedback on their measurements and training. For our target group, issues such as simple visualization, reporting of cut-offs and trends, and step-by-step instructions are important. A study of people with multimorbid chronic conditions also described how a simple digital representation of health data could help, as the graphs and tables they receive from their physicians are often not easy to understand [31]. A point also raised by our participants was the synchronization of measurements in a system and their interpretable presentation; this point was also discussed as very important by older people in a study of requirements for sensor-based frailty assessment at home [32].

Despite the general willingness and openness to the possibility of early detection of changes in nutritional and physical activity outcomes and targeted training, it was clear that human involvement was desired. The presumed desired level of involvement varied between the opinion that it would be sufficient if a qualified person introduced the user to the use of the MuTs and the opinion that independent use would not be possible without the constant presence of qualified staff. Therefore, the MuTs should be set up in a public place that is connected to the research facility or, for example, to a physiotherapy practice or a medical facility. Our findings reflect the findings of a scoping review of web-based interventions to promote healthy lifestyles in older adults. The review identified 5 papers that addressed the issue of support. Results of the review showed that although the need for human involvement was emphasized, the desired level of support varied from purely web-based support to a personal introduction to the system to face-to-face sessions with a trainer [34]. Similar results were found in a focus group study with older people on the use of an exergame telerehabilitation program. Participants also felt that human involvement and the possibility of human contact would be important to them [29]. Participants in our focus groups also discussed the desire for a means of contact and the interest in integrating an emergency system. The importance of human involvement in the use of automated measurement systems is also apparent in a qualitative study of the development of a home-based frailty assessment. Participants described concerns about the loss of personal contact with health care professionals when measurements were taken by sensors [32]. In our focus groups, participants also expressed that they would generally welcome a reduction in the burden on physicians and other health care professionals but that the station should rather be used for monitoring over time and that professional advice should be sought if there are signs of health problems, for example, by making an appointment with a physician. During the discussion on the collection of cardiological health data, it also became clear that measurements that are perceived as more complicated or demanding (such as ECG) should be carried out by a physician.

The results of our focus groups indicated that participants were less concerned about data protection and their privacy. Although it was discussed that it would be important for other participants not to be in the same room when completing questionnaires on the MuTs screen, there were no comments about the concerns about general data security or worries about unauthorized access to data collected in the station. In general, there seem to be different perceptions and needs of older people in terms of privacy and data security. While some studies using sensor-based systems in the home environment to collect health data seem to be more associated with concerns about these parameters, and there are also increased concerns in the context of using apps to support the recording of various disease-related symptoms [28,31,32], a study of exergame-based telerehabilitation also reported fewer concerns among participants about data security [29]. Interestingly, the study by Bian et al [28] also explicitly opposed the use of cameras. In our focus groups, participants were shown the analysis of exercise performance using 3D depth cameras, and different types of recording were also discussed, with the color image being the most popular. It is likely that a key difference between our study and the study by Bian et al [28] is that in the study by Bian et al [28], the cameras were installed in the participants’ home environment, and in our study, they were installed in the MuTs. This may suggest that, in this context, a more comprehensive collection of health data could be carried out with sensor technology that may be less accepted in the home environment for people who still have sufficient mobility to visit a MuTs.

The results of this study must be considered in the context of a number of limitations. No systematic sampling strategy was used, and the study was not stratified by age or sex. In particular, the second focus group in the rehabilitation center had fewer female participants than the actual proportion of women in this age group in the German population [35]. Recruitment in the rehabilitation center was particularly challenging: As inpatient geriatric rehabilitation in Germany usually lasts about 3 weeks, the focus group appointment had to be arranged quickly after the participants had given their consent and also had to be coordinated with the patients’ other therapy measures and appointments. In addition, 1 ward was closed during recruitment due to a COVID-19 outbreak, and patients from this ward could not be recruited. In addition, the main diagnosis of people in geriatric rehabilitation was not recorded. It is, therefore, possible that rehabilitants responded differently depending on their main diagnosis. This cannot be verified with the available data. In general, it cannot be ruled out that more homogeneous focus groups would have allowed for different responses and opinions. Moreover, it is also possible that the focus groups were composed of people who were generally more interested in nutrition, exercise, and technology.

### Conclusions

For older people with initial health limitations in particular, the regular use of a MuTs could be a promising approach to enable independent monitoring of nutrition and physical activity status. Important requirements and prerequisites for the development of a MuTs were collected, which will be developed and evaluated with the involvement of the target group. In particular, aspects such as the necessary level of supervision by a real person on site and the independent usability of several measuring and training devices in the context of using the MuTs should be given special consideration in further development.

## Appendix

Multimedia Appendix 1 Coding system for focus groups 1 to 3.

Multimedia Appendix 2 Coding system for focus groups 4 and 5.

Multimedia Appendix 3 Presented training options and list of discussed advantages and disadvantages of each training option.

## Abbreviations

COREQ: Consolidated Criteria for Reporting Qualitative Research
ECG: electrocardiography
MuTs: measurement and training station

## Data Availability

The data generated during this study are available from the corresponding author upon reasonable request.
