# Supplementary material for: Requirement Analysis of Different Variants of a Measurement and Training Station for Older Adults at Risk of Malnutrition and Reduced Mobility: Focus Group Study
Source: JMIR Aging. 2024 Sep 17;7:e58714. doi: 10.2196/58714 (PMC11445625; doi:10.2196/58714)
Supplement: Multimedia Appendix 1 [file aging_v7i1e58714_app1.docx]

Table 2: Codingsystem from focusgroup 1-3

| **Main Category** | **Subcategory** |
| --- | --- |
| Health data and measurements |  |
|  | Oxygen Saturation |
|  | Steps |
|  | Handgrip strength |
|  | Cardiological health data and measurements |
| Measuring devices |  |
|  | Handgrip strength measurement device |
|  | Fitnesstracker |
|  | Blood pressure monitor (wrist) |
|  | Blood pressure monitor (forearm) |
| Physical training variants in the measurement and training station |  |
|  | Sensomotor training device with cushioned oscillating unstable platform (here Posturomed) |
|  | Practise staircase |
|  | Cognitive-motor exergames (here Dividat Senso) |
|  | Exercise execution analysis using 3D depth imaging camera system |
|  | Bicycle ergometer |
| Range of use of the measuring and training station |  |
|  | Length of stay at the measuring and training station |
|  | Frequency of visits to the measuring and training station |
|  | Planning visits at the measuring and training station |
| Guided tour of the visit |  |
|  | Touchscreen use |
| Guidance/feedback on training & measurement |  |
|  | Feedback  Instructions |
