# Supplementary material for: Requirement Analysis of Different Variants of a Measurement and Training Station for Older Adults at Risk of Malnutrition and Reduced Mobility: Focus Group Study
Source: JMIR Aging. 2024 Sep 17;7:e58714. doi: 10.2196/58714 (PMC11445625; doi:10.2196/58714)
Supplement: Multimedia Appendix 2 [file aging_v7i1e58714_app2.docx]

Table 3: Codingsystem from focusgroups 4,5

| **Main Category** | **Subcategory** |
| --- | --- |
| Performing the handgrip strength measurement |  |
|  | Performing the measurement |
|  | Supportive aspects for implementing digital handgrip strength measurement |
|  | Expectations of handgrip strength measurement |
|  | Limited usability during execution |
| Evaluation of the hand grip strength measurement |  |
|  | Interpreting the shown results screens |
|  | Positive design aspects |
|  | Negative design aspects |
| Filling out questionnaires on a Touchscreen (Tablet or 55” screen) |  |
|  | Supportive factors for digital completion of questionnaires |
|  | Barriers for digital completion of questionnaires |
| Physical training variants in the measurement and training station |  |
|  | Sensomotor training device with cushioned oscillating unstable platform (here Posturomed) |
|  | Cognitive-motor exergames (here Dividat Senso) |
|  | Exercise execution analysis using 3D depth imaging camera system |
|  | Other training variants |
| Continuing exercises at home |  |
|  | Frequency with which exercises could be continued at home |
|  | Conditions and influencing factors for practicing at home with the tablet |
