# Supplementary material for: Requirement Analysis of Different Variants of a Measurement and Training Station for Older Adults at Risk of Malnutrition and Reduced Mobility: Focus Group Study
Source: JMIR Aging. 2024 Sep 17;7:e58714. doi: 10.2196/58714 (PMC11445625; doi:10.2196/58714)
Supplement: Multimedia Appendix 3 [file aging_v7i1e58714_app3.docx]

Table 4: Presented training options and list of discussed advantages and disadvantages of each training option.

|  | **Exercise stairs** | **Oscillatory Platform** | **Exergaming System** | **3D depth image-based training correction** | **Bicycle ergometer** |
| --- | --- | --- | --- | --- | --- |
| Round 1 | - Available everywhere - Real staircase is more attractive - Not usable due to reduced mobility - Relevance to everyday life - Variable step heights | - No exercise correction if exercise performed incorrectly - Too demanding on balance - Training focus on balance - Already known, if unknown then quite curious | - Cannot be used by those with severe physical limitations - Training focus on coordination, reaction, cognition - Playful, motivating - Instruction through device - Elsewhere not available | - No visible support options - Uncertainty about reliability/quality of correction - Direct feedback on exercise performance - Adaptability of exercises - Elsewhere not available | - Already in use - Outdoor cycling more attractive - Not usable due to certain illness - Low threshold activity - Already known - Positive experience with certain diseases |
| Ranking  n = 12 | **5**  (mean: 3.5) | **2**  (mean: 2.8) | **1**  (mean: 2.4) | **3** (mean: 3.1) | **4**  (mean: 3.3) |
| Round 2 | Not discussed  in focus group round 2 | - No feedback available - Training too demanding - Little variety - Simple principle - Positive expectation of training effectiveness | - Instant feedback - Versatile training options - Playful, exciting - Focus on coordination, reaction, cognition | - Seems complicated, extensive technology - Concerns about susceptibility to error - Direct feedback on exercise performance - Can be used by people with balance problems | Not discussed  in focus group round 2 |
| -: negative aspect, +: positive aspect,  Ranking: Participants rated the options presented on a scale of 1 to 5, with 1 being the most preferred and 5 being the least preferred option for MuTs. | | | | | |
